# Usefulness of surgical lung biopsies after cryobiopsies when pathological results are inconclusive or show a pattern suggestive of a nonspecific interstitial pneumonia

**DOI:** 10.1186/s12931-020-01487-w

**Published:** 2020-09-04

**Authors:** Benjamin Bondue, Dimitri Leduc, Antoine Froidure, Thierry Pieters, Olivier Taton, Vincent Heinen, Patrick Alexander, Delphine Hoton, Florence Dome, Myriam Remmelink

**Affiliations:** 1 Department of Pneumology, Hôpital Erasme, Université libre de Bruxelles, 808 route de Lennik, 1070 Brussels, Belgium; 2 Department of Pneumology, Cliniques Universitaires Saint-Luc, Université Catholique de Louvain, Brussels, Belgium; 3 Department of Pneumology, Centre Hospitalier Universitaire de Liège, Liège, Belgium; 4 Department of Pneumology, AZ Glorieux, Ronse, Belgium; 5 Department of Pathology, Cliniques Universitaires Saint-Luc, Université Catholique de Louvain, Brussels, Belgium; 6 Department of Pathology, Centre Universitaire de Liège, Liège, Belgium; 7 Department of Pathology, Hôpital Erasme, Université libre de Bruxelles, Brussels, Belgium

**Keywords:** Cryobiopsy, Surgical lung biopsy, Interstitial lung disease, Diffuse parenchymal lung disease, Trans-bronchial lung cryobiopsy, Nonspecific interstitial pneumonia, NSIP, Idiopathic pulmonary fibrosis, IPF

## Abstract

**Background:**

Although increasing data supports the use of transbronchial lung cryobiopsies (TBLCs) for the diagnosis of diffuse parenchymal lung diseases (DPLDs), its role as an alternative to surgical lung biopsy (SLB) is still under debate. The aim of this study was to assess the benefit of additional SLBs performed in selected patients after TBLCs.

**Method:**

We conducted a multicentric Belgian prospective trial in which SLBs were performed after TBLCs when the pathological diagnosis was uncertain or if a nonspecific interstitial pneumonia (NSIP) pattern was observed hypothesizing that SLB could provide additional information and that a co-existent UIP pattern could be missed.

**Results:**

Eighty-one patients with TBLCs performed for a DPLD were included in the study between April 2015 and December 2019. A specific histological diagnosis was obtained in 52 patients (64%) whereas no pathological diagnosis following TBLCs was obtained in 13 patients (16%) and a pattern suggestive of a NSIP was observed in 16 patients (20%). Fourteen out of these 29 patients had SLBs after TBLCs. SLBs showed a UIP pattern in 11 (79%), a pattern suggestive of a hypersensitivity pneumonitis in two (14%) and a NSIP pattern in one patient (7%). Among the 16 patients with pathological NSIP following TBLCs, six underwent a SLBs showing a UIP in five and confirming a NSIP in one patient only. A retrospective pathological analysis of patients having both procedures showed a lower diagnostic confidence and agreement among pathologists for TBLCs compared to SLBs. Major factors underlying the added value of SLBs were the bigger size of the sample as well as the subpleural localization of the biopsies.

**Conclusions:**

TBLCs are useful in the setting of DPLDs with a good diagnostic yield. However, our study suggests that SLB provides critical additional information in case TBLCs are inconclusive or show a pattern suggestive of a NSIP, questioning the accuracy of TBLC to adequately identify this histological pattern.

## Introduction

Diffuse parenchymal lung diseases (DPLDs) are a heterogeneous group of diseases with a variable amount of fibrosis and inflammation. For prognostic and therapeutic purposes, a precise diagnosis is required. The whole diagnostic process should be performed and discussed within an experienced team during a multidisciplinary discussion (MDD) [1, 2]. When the diagnosis based on clinical, radiological and biological data is inconclusive, a lung biopsy is recommended [1]. Surgical lung biopsies (SLBs) are currently considered as the gold standard for this purpose as stated in idiopathic pulmonary fibrosis (IPF) guidelines [1].

However, SLB is an invasive procedure with significant comorbidities, a hospitalization for a few days, systematic chest drainage, and a postoperative mortality rate of 2% up to 3.6% [3–7]. Therefore, trans-bronchial lung cryobiopsy (TBLC) is increasingly recognized as an alternative technique for the diagnosis of DPLDs [6, 8–11]. TBLC appears to be safer than SLB, and its contribution to the diagnosis obtained via multidisciplinary discussion is comparable to that of SLB, although the histological diagnostic yield is higher with SLB (approximately 80% for TBLC vs 95% for SLB) [11].

Interestingly, whereas many data support the use of TBLC for the diagnosis of DPLDs, only three studies compared the result of TBLC and SLB performed in a same patient. A first study was conducted on a limited number of patients (seven) [12] while the other two were conducted on a larger cohort, both providing conflicting results concerning the agreement between TBLC and SLB for histological diagnoses [13, 14]. Nevertheless, the COLDICE study [14] was the biggest and the unique multicentric study showing a high level of agreement between TBLC and SLB for both histopathological interpretation and MDD diagnoses. In these studies, limited data are available concerning nondiagnostics TBLCs or TBLCs showing a pattern suggestive of a NSIP, two situations in which SLB could particularly provide substantial additional information. Idiopathic NSIP that is nowadays considered as a specific entity [2], is indeed defined histologically by variable proportions of interstitial inflammation and fibrosis with a uniform appearance [15]. However, the assessment of this pathological uniformity is based on the analysis of SLBs that are bigger than TBLCs and performed in different lobes.

Consequently, we hypothesized that the accuracy of NSIP diagnosis obtained on smaller biopsies such as cryobiopsies could be lower than on SLB [9]. NSIP pattern could be mistakenly recognized as other important features could be observed in SLB samples. Particularly, the spatial heterogeneity, important for the diagnosis of UIP patterns, could be missed on TBLCs [16]. This misinterpretation is particularly important as it can have prognostic and therapeutic consequences.

Therefore, the added value of SLB performed after TBLC was evaluated in this study when the latter showed nondiagnostic or unspecific pathological results without confident diagnosis following another multidisciplinary discussion. Preliminary results obtained on a limited number of patients were published in 2017 [9]. In this follow up study, we provide more evidence supporting the benefit of performing SLBs in these selected situations. Moreover, a blinded retrospective analysis of paired TBLCs and SLBs was conducted by three experienced pathologists in ILDs to determine the differences existing between both sampling techniques and to compare the agreement among pathologists for both types of biopsies.

## Material and methods

### Study design

A multicentric prospective observational study was performed between April 2015 and December 2019 in three Belgian academic hospitals. The study was approved by the ethical committees of the non-leading hospitals and by the leading ethical committee of the Erasme hospital (ref P2015/192). Patients were included if a lung biopsy was indicated and validated by a MDD for the diagnosis of a DPLD. The primary endpoint of the study was to determine the benefit of performing SLBs when TBLCs showed unspecific results (including a pattern suggestive of an NSIP) and without a confident diagnosis following another MDD. Secondary endpoints were to determine the diagnostic yield of TBLC for the diagnosis of DPLDs, to determine the rate of complications related to TBLCs, and to perform a blinded and retrospective review of the paired SLBs and TBLCs samples by the pathologists from the three participating centers. From this retrospective part of the study, the main determinants underlying the hypothesized difference between SLB and TBLC samples will be highlighted, as well as the diagnostic concordance among the three pathologists for both sampling techniques and the level of confidence of the histological diagnosis between paired SLBs and TBLCs samples.

The composition of the multidisciplinary teams includes at least one chest physician, one pathologist, one thoracic radiologist, one specialist in internal medicine or rheumatologist. The patients were informed of the possibility to have a surgical biopsy following the endoscopic procedure in case of equivocal pathological diagnosis or histopathologic pattern suggestive of NSIP. SLBs were obtained by video-assisted thoracoscopic surgery (VATS) and performed in at least two different lobes as recommended in the guidelines for patients with suspected idiopathic pulmonary fibrosis (IPF) [1]. Of note, MDD was conducted in each participation center without blinding, and all indications and results of biopsies (both SLBs and TBLCs) were discussed within the multidisciplinary team. A SLB was not performed in patients with a histological NSIP pattern following TBLCs if an associated diagnosis (such as a hypersensitivity pneumonitis) could be made by the MDD with a reasonable probability according to the analysis of all the available data. This includes the HRCT pattern, cellularity of the bronchoalveolar lavage, specific IgG, autoantibodies, drugs, environmental and occupational exposures.

### Study population

Written informed consent for participation in the study was obtained from each patient. Inclusion criteria were the followings: patients at least aged of 18 years old with a pulmonary systolic arterial pressure estimated by echocardiography of less than 40 mmHg. Exclusion criteria included the presence of a coagulopathy (platelet count < 100,000/mm^3^, prothrombin time international normalized ratio - INR > 1.5, activated partial thromboplastin time - APTT > 35), hypoxemia (PaO_2_ < 55 mmHg on room air) or hypercapnia (PaCO_2_ > 45 mmHg), and severe underlying cardiac disease. Collagen vascular disease-associated interstitial lung disease (CVD-ILD) and drug-induced interstitial lung disease (D-ILD) were not formally excluded but efforts were done to avoid lung biopsies in such conditions as the diagnosis can be achieved by other means.

### Bronchoscopy and cryobiopsy

Procedures were performed as previously described [9]. Briefly, all procedures were performed under general anesthesia. We attempted to obtain four biopsies from two different segments of the most affected lobe. For each biopsy, the cryoprobe (1,9 or 2,4 mm, Erbe, Germany) was pushed under fluoroscopic guidance to the distal parenchyma and the probe was withdrawn of 1-2 cm from the thoracic wall. To control potential severe bleedings, a Fogarty balloon was systematically placed in the lobar bronchus close to the sampled segment, and inflated immediately after biopsy. The bleeding was scored 0 if no bleeding, 1 (mild bleeding) if bleeding stopped with aspiration only and/or insufflation of the Fogarty balloon less than 5 min, 2 (moderate bleeding) if cold saline was used to control the bleeding and/or the Fogarty balloon need to be inflated more than 5 min, and 3 (severe bleeding) if any of the following treatment was required: embolization, selective bronchial intubation, transfusion, admission in an intensive care unit (ICU), or resulting in death or prolonged hospitalization.

### Biopsy specimens

Biopsy specimens were fixed in 10% formalin and embedded in paraffin. Hematoxylin and eosin as well as Masson’s Trichrome, Giemsa, staining were performed as well as immunostaining against pancytokeratins.

### Pathological review

A retrospective histological analysis was performed for patients having both biopsies (TBLCs and SLBs). Three experienced pathologists in DPLDs (MR, FD, and DH) reviewed all biopsies. Biopsies were anonymized (each sample receiving a code number). For each specimen and pathologist, the most likely histological diagnosis and its level of confidence (high, low or very low) were recorded. Histopathological patterns were recognized following the 2018 guideline-refined categories of definite or probable usual interstitial pneumonia, indeterminate for usual interstitial pneumonia, or alternative diagnosis [1]. For some biopsies, no pathological diagnosis could be identified. In that case, the diagnostic confidence was categorized as “not applicable”. Histological patterns of probable or indeterminate for UIP were categorized as UIP with a low level of confidence.

Then, a comparison of TBLCs and corresponding SLBs of each patient was performed by each pathologist to evaluate whether the SLB provided additional information as well as to identify the underlying reasons (including the size and the localization of the biopsies). The agreement among pathologists for TBLCs and SLBs diagnoses and the corresponding level of confidence were also determined and compared.

### Statistical analysis

According to the results of the D’Agostino & Pearson test used to assess the normality of the sample values, simple comparisons between two groups were tested by unpaired Student t tests or Mann-Whitney tests. Proportions were compared using the Chi^2^ test. Statistical analyses were performed using GraphPad Prism 6 (GraphPad Software, La Jolla, California, USA). For all tests, a *P*-value of less than 0.05 was considered statistically significant.

## Results

### TBLCs are useful for the diagnosis of DPLDs

**Fig. 1:**
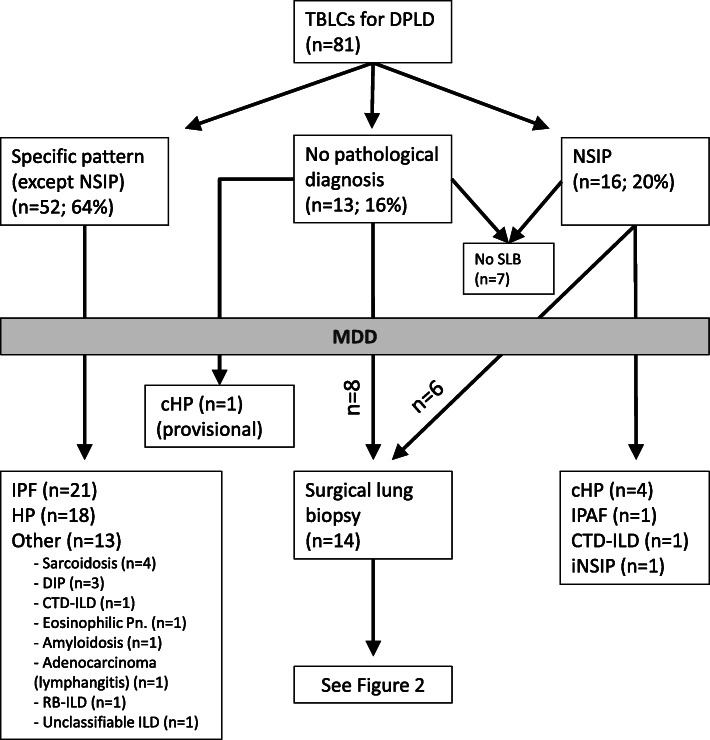
Flow diagram of the main results obtained for the 81 patients included in the study and having TBLCs for the diagnosis of a DPLD. cHP: chronic hypersensitivity pneumonia; CTD-ILD: connective tissue disease-associated interstitial lung disease; DIP: desquamative interstitial pneumonia; DPLD: diffuse parenchymal lung disease; iNSIP: idiopathic non-specific interstitial pneumonia; IPAF: interstitial pneumonia with auto-immune features; MDD: multidisciplinary discussion; RB-ILD: respiratory bronchiolitis-associated interstitial lung disease; SLB: surgical lung biopsy; NSIP: non-specific interstitial pneumonia; TBLC: trans-bronchial lung cryobiopsy

81 patients with TBLCs performed for the diagnosis of a DPLD were included between April 2015 and December 2019. The main clinical characteristics and detailed pathological diagnoses are summarized in the Table 1. Four TBLCs were performed in the most affected lobe with a mean size of 21 mm^2^ (ranging from 9 to 44 mm^2^) (Table 1). The preservation of TBLC samples was good to excellent without freezing-related cellular alterations. A specific pathological pattern (excluding a NSIP pattern) was observed in 52 patients (64%) whereas undetermined or NSIP patterns were observed in 13 (16%) and 16 (20%) patients respectively (Fig. 1).

**Table 1:** Characteristics of the patients included in the study and having TBLCs performed for the diagnosis of a DPLD

		Overall population (***n*** = 81)	Specific histological pattern (***n*** = 52)	NSIP or nondiagnostic pattern (***n*** = 29)	***p*** value
Gender	Male N (%)	40 (49)	28 (54)	12 (41)	NS
Age, years	Median (range)	62 (26–81)	62 (26–81)	64 (32–75)	NS
Smoking history	Current N (%)	11 (14)	7 (13)	4 (14)	NS
	Former N (%)	43 (53)	28 (54)	15 (52)	NS
	Never N (%)	27 (33)	17 (33)	10 (34)	NS
BMI	Median (range)	28 (17–39)	27 (17–39)	29 (18–39)	NS
FVC, % predicted value	Median (range)	78 (40–134)	78 (40–130)	79 (41–134)	NS
DLCO, % predicted value	Median (range)	52 (20–85)	52 (20–79)	48 (28–85)	NS
HRCT	Typical UIP N (%)	1 (1)	1 (2)	0 (0)	NS
	Probable UIP N (%)	9 (11)	5 (10)	4 (14)	NS
	Indeterminate for UIP N (%)	30 (37)	19 (37)	11 (38)	NS
	Alternative diagnostic N (%)	41 (51)	27 (52)	14 (48)	NS
Number of biopsies/patient	Mean (range)	4 (1–5)	4 (2–5)	4 (1–5)	NS
Size of biopsies (mm2)	By specimen Mean (range)	20,9 (9–44)	22 (10–44)	18 (9–40)	0.049
	By patient Mean (range)	76,5 (30–152)	82 (39–152)	65 (30–120)	0.007

Patients were categorized according to the result of the TBLC (specific histological pattern or non-diagnostic/NSIP pattern). Comparisons between groups were made using unpaired Student t tests or the Chi^2^ test (proportions). A *P*-value of less than 0.05 was considered statistically significant

Complications following TBLCs were mainly hemorrhage (mild or moderate in most of the cases) and pneumothoraxes (21%, half of them requesting a chest drainage) (Table 2). A severe bleeding was present in four patients (5%) and required at least a prolonged hospitalization and an ICU admission for a 24-h monitoring. No recurrence of bleeding was observed. Whereas the bleeding was controlled in three out of the four patients by a prolonged use of the Fogarty balloon and cold saline solution, one patient required a selective bronchial intubation. In this patient, an acute exacerbation also occurred during the prolonged hospitalization, resulting in an important worsening of the respiratory condition, which required a lung transplantation 2 months after.

**Table 2:** Complications, diagnostic yield and histological diagnosis after TBLC (*N* = 81)

Hemorrhage N (%)	Grade 0	4 (6)
	Grade 1 (mild)	47 (58)
	Grade 2 (moderate)	25 (31)
	Grade 3 (severe)	4 (5)
Pneumothorax N (%)	All	17 (21)
	Requiring chest drainage	9 (11)
Diagnostic yield N (%)		60 (74)
Specific histological diagnosis N (%)	Including NSIP	68 (84)
	Excluding NSIP	52 (64)
Detailed histological diagnosis following TBLCs N (%)	UIP	21 (26)
	HP	18 (22)
	NSIP	16 (20)
	Undetermined	13 (16)
	Sarcoidosis	4 (5)
	DIP	3 (4)
	NSIP/COP overlap	2 (2)
	Amyloidosis	1 (1)
	Eosinophilic pn.	1 (1)
	RB-ILD	1 (1)
	Adenocarninoma (lymphangitis)	1 (1)

Comparison of patient’s characteristics between those with nondiagnostic TBLCs and the others showed no differences in term of HRCT pattern distribution but a significant difference in term of size of the cryobiopsies. Indeed, the total surface area per patients of nondiagnostic specimens was smaller than those given a specific diagnosis (65 ± 24 vs 82 ± 27 mm^2^, mean ± SD; *p* < 0,01) (Table 1). We further determined that with a cut-off of a total surface area of 110 mm^2^, the risk to have nondiagnostic biopsies was lower than 5%. In line with this observation, two patients had only one TBLC (instead of the attempted four) and none of them had a specific histological diagnosis.

### SLB provides critical additional information in case TBLCs are inconclusive or show a pattern suggestive of a NSIP

**Fig. 2:**
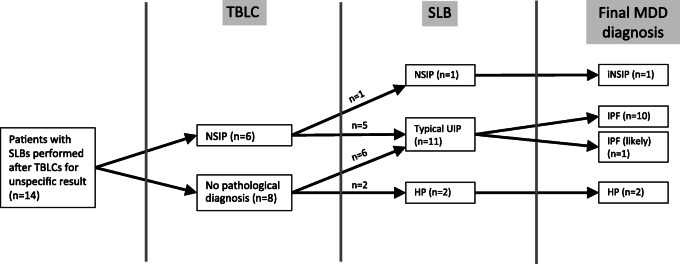
Pathological and final MDD diagnoses obtained in the 14 patients having both TBLCs and SLBs

Among the 29 patients without specific diagnosis following TBLCs, seven did not consent to have a SLB (further classified as unclassifiable ILD), and eight received a provisional diagnosis following a novel MDD (5 chronic HP, 2 IPAF, 1 idiopathic NSIP). The reasons for retrieving consent to have a SLB are detailed in supplemental data (Table S1). A SLB was performed for the other 14 patients (six with a suspected NSIP pattern and eight without pathological diagnosis following TBLCs) (Fig. 2). Complications of SLBs within the first 3 months following the biopsies were an acute exacerbation (*n* = 1) and a transient atrial fibrillation (n = 1). No SLB-related death was reported and the median hospitalization time was 5 days (4 days for the chest drainage). As expected, SLB provides ten-fold bigger sample than TBLC (76 ± 27 mm^2^ for TBLCs vs 676 ± 307 mm^2^ for SLB, mean ± SD) and subpleural area was always present for pathological examination (Supplemental figure, Figure S1). In 11 out of the 14 patients having both TBLC and SLB, a UIP pattern (10 typical and one probable) was observed in the SLB (79%). Therefore, even if the HRCT pattern was mostly indeterminate or suggestive of another diagnosis in one patient, the final MDD diagnosis was an IPF in 10 and an IPF “likely” in one patient according to the current guidelines (Fig. 2) [1]. Interestingly, a NSIP pattern was only confirmed in 1 out of the 6 patients with a suspected NSIP on TBLC (17%), and a HP pattern was observed in two other patients without pathological diagnosis following TBLC. Based on the final MDD taking into account SLB data, the final diagnosis changed in 93% of the patients having both types of biopsy (83% of those with a NSIP pattern on TBLC). In other words, our data showed that if a SLB is performed in a patient with a NSIP pattern on TBLC, another diagnosis will be observed in 83% of the cases changing significantly the final diagnosis and potentially the subsequent treatment.

Considering patients with specific histological diagnoses provided by the TBLCs, patients with NSIP patterns on TBLCs for which sufficient clinical, biological and radiological data allow the MDD to make a diagnosis without SLBs, and the patient with a NSIP pattern confirmed with both sampling techniques, the diagnostic yield of TBLCs was 74% (Table 2).

### Inter-observer agreement and diagnostic confidence are higher for SLBs than TBLCs

**Fig. 3:**
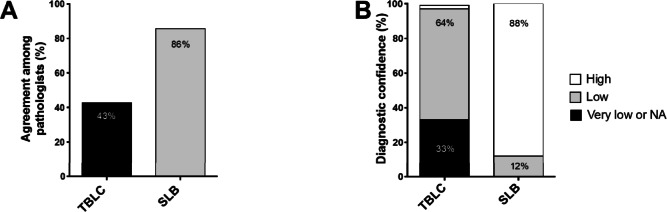
Comparison between TBLC and SLB regarding the percentage of agreement among pathologists for the most likely diagnosis (**a**), and the corresponding level of confidence (**b**). NA: not applicable (non-diagnostic biopsy)

Three ILDs experienced pathologists further retrospectively and blindly reviewed all biopsies from the 14 patients who benefited from both procedures. Each pathologist was asked to define the most likely diagnosis, even with a low confidence level. Major determinants underlying differences between TBLCs and SLBs were depicted. This analysis demonstrated a higher diagnostic concordance among pathologists for SLB (86%) compared to TBLCs (43%) (Fig. 3). Moreover, the level of confidence of the histological diagnosis was much higher for SLBs than for TBLCs (high diagnostic confidence in 88% of SLB contrasting with low and very low level of confidence in 97% of TBLCs) (Fig. 3). The benefit to perform SLB after TBLC in this selected population was not only to improve the diagnostic confidence as it changed the final diagnosis in 60% of the cases in the retrospective pathological analysis (Table 3). Accordingly, the overall agreement between paired TBLC and SLB for specific histopathological pattern was 40%. When SLBs provide additional information (change in the histological diagnosis and/or an increase in the level of confidence), the major underlying determinants were the size of the biopsies in 90%, a better representation of the subpleural area in 50% and the localization of the biopsies in different lobes in 23% of these cases.

**Table 3:** Results of the pathological review of paired TBLCs and SLBs samples by the three experienced pathologists in ILD

		Pathologist			Mean (SD)
		MR	DH	FD	
**Change in histological diagnosis following SLB (excluding limited changes in confidence level) % (N/Number of patients with TBLC and SLB)**		64 (9/14)	57 (8/14)	57 (8/14)	**60 (4)**
**Additional informations provided by SLB (including changes in confidence level) % (N/Number of patients with TBLC and SLB)**		93 (13/14)	71 (10/14)	79 (11/14)	**81 (11)**
Major determinants	Localisation of the biopsy in different lobes	23 (3/13)	10 (1/10)	36 (4/11)	**23 (13)**
	Size of the biopsies	69 (9/13)	100 (10/10)	100 (11/11)	**90 (18)**
	Localisation (subpleural vs bronchiolocentric)	46 (6/13)	30 (3/10)	73 (8/11)	**50 (22)**

Each pathologist (MR, DH, and FD) provides the most probable histological diagnosis for the 14 patients having both SLBs and TBLCs. For each patient and pathologist, change in histological diagnosis was recorded (row 1) as well as change in histological and/or confidence level (row 2). When SLB provide additional information (including only changes in confidence level), it was asked to the pathologist to describe the main reasons (row 3)

Finally, considering the identification of a NSIP pattern on TBLC, the three pathologists retrospectively analyzed the samples of the six patients having TBLCs showing a NSIP pattern and a corresponding SLB. If the concordance between pathologists was perfect to identify a pattern of NSIP, each of them should have identified six NSIP patterns during this retrospective analysis. Surprisingly, there was only an agreement among the pathologists to identify a NSIP pattern for two patients. Of note, its identification for any of the pathologist was systematically proposed without a high diagnostic confidence on TBLCs (Table 4). Moreover, the SLBs performed in these two patients only confirmed a NSIP pattern for one pathologist whereas another pathologist proposed a NSIP on SLBs but not on the corresponding TBLCs (Table 4).

**Table 4:** Identification of the NSIP pattern by the pathologists

	Pathologist		
	MR	DH	FD
NSIP on TBLC	5/14 (36%)	2/14 (14%)	3/14 (21%)
Level of confidence (n)	Indeterminate (2)	Low (1)	Indeterminate (1)
	Low (3)	Very low (1)	Very low (2)
NSIP on SLB	1/14 (7%)	0/14 (0%)	1/14 (7%)
NSIP on TBLC and SLB	1/5 (20%)	0/2 (0%)	0/3 (0%)

The three experienced pathologists (MR, DH, and FD) retrospectively reviewed samples for which both TBLCs and SLBs were available (*n* = 14). Recognition of a NSIP pattern was recorded as well as its level of confidence

## Discussion

Increasing data support the use of TBLCs for the diagnosis of DPLDs with a diagnostic yield ranging between 72 and 87% [11]. In line with these published results, a diagnostic yield of 74% was observed from the 81 patients included in this study. The rate of pneumothorax (21%) was relatively high compare to other reports. This is mostly explained by a high prevalence of fibrotic DPLDs (46%) known to be associated with higher risk of pneumothorax [10]. Bleeding was mild to moderate in a majority of the cases. There was no TBLC related death. Altogether, our data support that TBLC are safe and globally useful for the diagnosis of DPLD used within MDD. Although no clear standards for adequate sampling in TBLC exist, our data highlight that sample size is of utmost importance. Indeed, non-diagnostic TBLCs were frequently associated with smaller samples. Conversely, if the total surface area of TBLCs was higher than 110 mm^2^, the risk to have non-diagnostic biopsies was lower than 5%. The number of samples could also influence the frequency of non-diagnostic TBLCs (even if no statistical differences were noticed in our study between the groups). Indeed, more unspecific results were recorded in patients with only one sample. However, the number of patients in this subgroup was extremely low (*n* = 2) to draw definite conclusions.

Beside the growing evidence supporting the use of TBLCs for the diagnosis of DPLDs, only three studies directly compared TBLC and SLB performed in a same patient [12–14]. Romagnoli and colleagues included 21 patients and found a poor agreement for TBLC and SLB histological diagnoses [14]. This result contrasts with data from the recent and bigger COLDICE study including 65 patients and showing a histopathological agreement between TBLC and SLB of 71%. Moreover, for TBLC with a high to definite confidence at MDD, 95% of TBLC diagnoses were concordant with SLB diagnoses [13]. In these studies, SLB are performed in all patients whatever the level of confidence obtained after TBLC (5% of non-diagnostics TBLCs in the COLDICE study). Moreover, these studies do not specifically address the accuracy of histological NSIP diagnosis in TBLCs compared to SLB (NSIP was only present on TBLC pathological analysis in 2 patients in the COLDICE study and in 3 patients in the Romagnoli study) [13]. In the present study, even if we acknowledge that the number of patients remains low, the benefit to perform SLB after TBLCs (following MDD) was specifically assessed in case of unspecific results including NSIP patterns on TBLC. It was prospectively founded that SLB provides additional information in 93% of the cases. Our results contrast therefore with those from the COLDICE study in which SLB provides additional information in 23% of the patients with low confidence or unclassifiable TBLCs diagnoses. Interestingly, NSIP pattern was only confirmed in 1 of the 6 patients with a suspected NSIP pattern on TBLC (17%) highlighting that a NSIP diagnosis should be questioned if based on TBLCs data only. Even if studies showed that TBLCs affect diagnostic confidence to a similar extend as SLBs within the context of MDD [17], our data showed that SLBs performed after TBLCs do not only increase the diagnostic confidence but also completely change the final diagnosis in most of the cases in this selected population of patients with unspecific TBLCs.

Finally, three pathologists conducted a retrospective and blinded analysis of pathological samples in order to better determine the differences between these two types of biopsies. This analysis does not reflect clinical routine but was interesting as it highlighted a higher inter-observer agreement among pathologists with higher confidence levels for SLBs diagnoses compared to TBLCs diagnoses. Moreover, the overall histopathological agreement (independently of the level of confidence) between paired TBLC and SLB was 40%, contrasting to the agreement observed in the COLDICE study of 71% [13]. Concerning the identification of a NSIP pattern, the diagnostic confidence was systematically low on TBLCs samples and confirmed on SLB in a minority of cases. Determinants underlying additional information from SLBs were analyzed. The bigger size of surgical biopsies was reported in a majority of the cases (90%) followed by differences in the localization of the biopsy (subpleural vs bronchiolocentric). Interestingly, the benefit to have samples from different lobes was only noted by the pathologists in only 23% of the cases.

Important limitations have to be addressed regarding the study. The number of patients having both procedures is small. Hence, additional studies from other groups and with a higher number of patients are required before drawing definite conclusions. In addition, multidisciplinary discussions were not performed blindly and by a unique multidisciplinary team (each of the three participating centers having its own team). Moreover, SLBs were not performed in all patients with TBLCs showing either a NSIP pattern or inconclusive results, representing another possible limitation. This was explained by a proportion of patients who denied having SLBs after TBLCs (24%) and by patients for which the MDD concluded to a diagnosis with an acceptable level of confidence (this possibility was prespecified in the protocol). Finally, nondiagnostic TBLCs were significantly smaller than those providing a specific diagnosis and the benefit to perform new TBLCs rather than SLBs in those patients was not evaluated.

## Conclusions

Although our study globally supports the role of TBLCs as an alternative to SLB for the multidisciplinary diagnosis of DPLDs, our results highlight the benefit to perform SLB in case of nondiagnostic TBLCs or when a pattern suggestive of a NSIP is observed. Our results also emphasize the importance of adequate sampling technique and sample size to limit the number of TBLCs with unspecific results.

## Supplementary information

**Additional file 1: Table S1.** Characteristics and main reasons of the seven patients to deny having SLBs after TBLCs. Three of the seven patients experienced a prolonged hospitalization following TBLC (one related to a pneumothorax, one related to a severe bleeding, and one secondary to both a severe bleeding and an acute exacerbation of the underlying ILD). All of the seven patients were afraid of adverse events following a SLB, denied to have another general anesthesia and preferred to have a follow up and/or a treatment even if their diagnosis remained uncertain. Of note, except patient #6 who underwent an acute exacerbation post TBLC, there was no absolute medical contra-indication to perform the SLB in the other six patients.

**Additional file 2: Figure S1** Illustration of the bigger size of SLB compared to TBLC. The pleura is also extensively present in the SLB (marked by the black arrow) and conversely most of the time absent in TBLC samples. Hematoxylin and eosin staining.

## Data Availability

The datasets used and/or analyzed during the current study are available from the corresponding author on reasonable request.

## Abbreviations

APTT: Activated partial thromboplastin time
BMI: Body mass index
cHP: Chronic hypersensitivity pneumonia
CTD-ILD: Collagen tissue disease-associated interstitial lung disease
CVD-ILD: Collagen vascular disease-associated interstitial lung disease
D-ILD: Drug-induced interstitial lung disease
DIP: Desquamative interstitial pneumonia
DLCO: Diffusion capacity for carbon monoxide
DPLD: Diffuse parenchymal lung disease
FVC: Forced vital capacity
HP: Hypersensitivity pneumonitis
HRCT: High resolution computed tomography
ICU: Intensive care unit
INR: Prothrombin time international normalized ratio
iNSIP: Idiopathic non-specific interstitial pneumonia
IPAF: Interstitial pneumonia with auto-immune features
IPF: Idiopathic pulmonary fibrosis
MDD: Multidisciplinary discussion
NSIP: Nonspecific interstitial pneumonia
RB-ILD: Respiratory bronchiolitis-associated interstitial lung disease
SLB: Surgical lung biopsy
TBLC: Transbronchial lung cryobiopsy
UIP: Usual interstitial pneumonia
VATS: Video-assisted thoracoscopic surgery
