# Supplementary material for: Usefulness of surgical lung biopsies after cryobiopsies when pathological results are inconclusive or show a pattern suggestive of a nonspecific interstitial pneumonia
Source: Respir Res. 2020 Sep 4;21:231. doi: 10.1186/s12931-020-01487-w (PMC7487918; doi:10.1186/s12931-020-01487-w)
Supplement: Supplementary file 1 — Additional file 1: Table S1. Characteristics and main reasons of the seven patients to deny having SLBs after TBLCs. Three of the seven patients experienced a prolonged hospitalization following TBLC (one related to a pneumothorax, one related to a severe bleeding, and one secondary to both a severe bleeding and an acute exacerbation of the underlying ILD). All of the seven patients were afraid of adverse events following a SLB, denied to have another general anesthesia and preferred to have a follow up and/or a treatment even if their diagnosis remained uncertain. Of note, except patient #6 who underwent an acute exacerbation post TBLC, there was no absolute medical contra-indication to perform the SLB in the other six patients. [file 12931_2020_1487_MOESM1_ESM.docx]

| **Patient** | **TBLC diagnosis** | **Complication of TBLC** | **Concerns about adverse events due to SLB** | **Willing to have a follow up or a treatment without a definite ILD diagnosis** |
| --- | --- | --- | --- | --- |
| #1 | Cellular NSIP | Pneumothorax requiring a chest drainage | Yes | Yes |
| #2 | Cellular NSIP | No | Yes | Yes |
| #3 | Unspecific | No | Yes | Yes |
| #4 | Unspecific | Severe bleeding  (grade 3) | Yes | Yes |
| #5 | Unspecific | No | Yes | Yes |
| #6 | NSIP + COP | Severe bleeding (grade 3) followed by an acute exacerbation | Yes. SLB contra-indicated due to the enhanced respiratory failure following TBLC and acute exacerbation | Yes |
| #7 | Unspecific | No | Yes | Yes |
