# Supplementary figures and images for: Usefulness of surgical lung biopsies after cryobiopsies when pathological results are inconclusive or show a pattern suggestive of a nonspecific interstitial pneumonia
Source: Respir Res. 2020 Sep 4;21:231. doi: 10.1186/s12931-020-01487-w (PMC7487918; doi:10.1186/s12931-020-01487-w)

## Slide 1
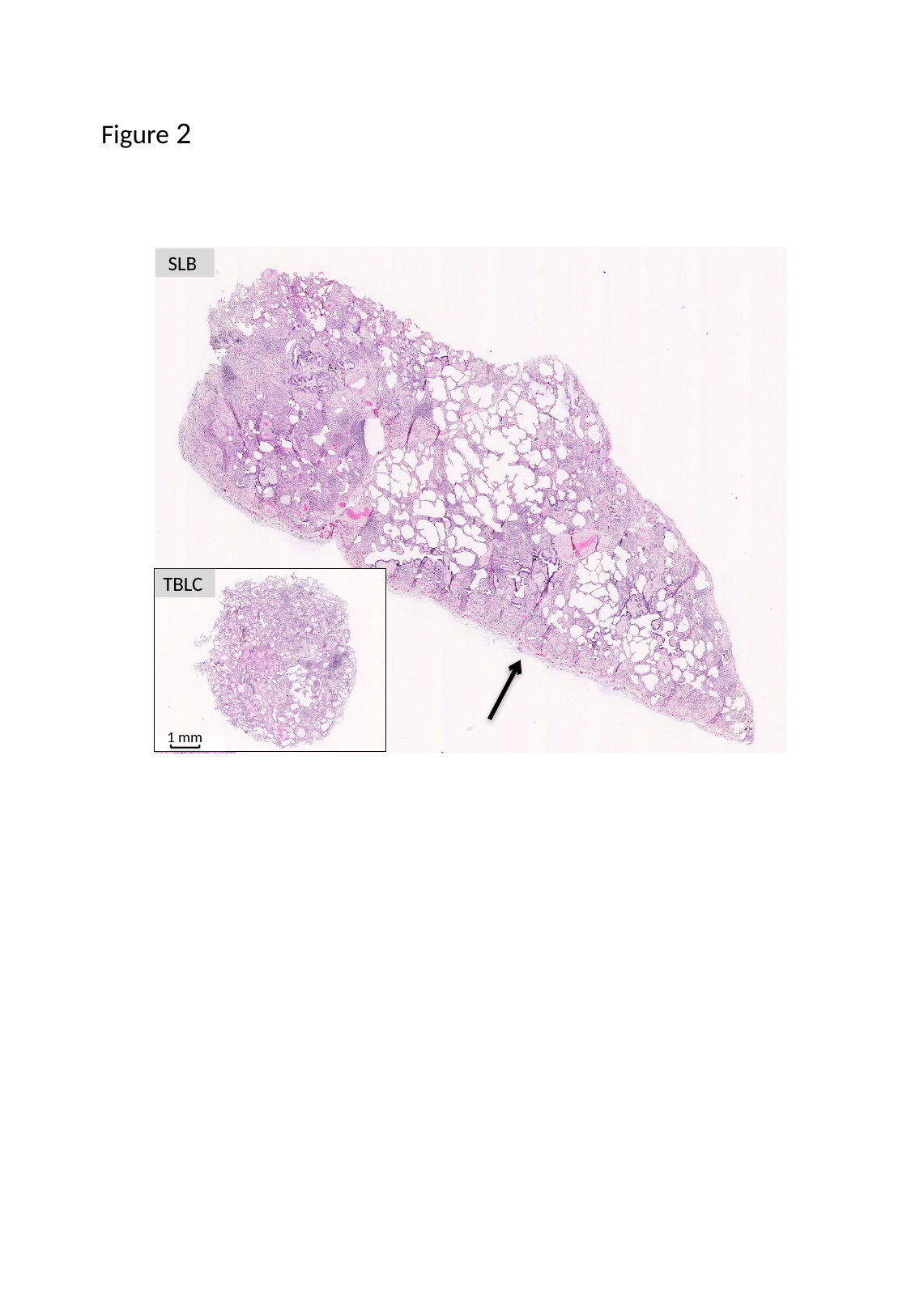

Figure 2
SLB
TBLC
1 mm

Supplement: Supplementary file 2 — Additional file 2: Figure S1 Illustration of the bigger size of SLB compared to TBLC. The pleura is also extensively present in the SLB (marked by the black arrow) and conversely most of the time absent in TBLC samples. Hematoxylin and eosin staining. [file 12931_2020_1487_MOESM2_ESM.pptx]
